# *Thermoanaerobacterium thermosaccharolyticum* β-glucosidase: a glucose-tolerant enzyme with high specific activity for cellobiose

**DOI:** 10.1186/1754-6834-5-31

**Published:** 2012-07-11

**Authors:** Jianjun Pei, Qian Pang, Linguo Zhao, Song Fan, Hao Shi

**Affiliations:** 1 College of Chemical Engineering, Nanjing Forestry University, Nanjing, 210037, China; 2 Jiangsu key Lab of Biomass Based Green Fuels and Chemicals, Nanjing, China

**Keywords:** β-glucosidase, Glucose tolerance, *Thermoanaerobacterium thermosaccharolyticum*, Over-expression, Phylogeny

## Abstract

**Background:**

β-Glucosidase is an important component of the cellulase enzyme system. It does not only participate in cellulose degradation, it also plays an important role in hydrolyzing cellulose to fermentable glucose by relieving the inhibition of exoglucanase and endoglucanase from cellobiose. Therefore, the glucose-tolerant β-glucosidase with high specific activity for cellobiose might be a potent candidate for industrial applications.

**Results:**

The β-glucosidase gene *bgl* that encodes a 443-amino-acid protein was cloned and over-expressed from *Thermoanaerobacterium thermosaccharolyticum* DSM 571 in *Escherichia coli*. The phylogenetic trees of β-glucosidases were constructed using Neighbor-Joining (NJ) and Maximum-Parsimony (MP) methods. The phylogeny and amino acid analysis indicated that the BGL was a novel β-glucosidase. By replacing the rare codons for the N-terminal amino acids of the target protein, the expression level of *bgl* was increased from 6.6 to 11.2 U/mg in LB medium. Recombinant BGL was purified by heat treatment followed by Ni-NTA affinity. The optimal activity was at pH 6.4 and 70°C. The purified enzyme was stable over pH range of 5.2–7.6 and had a 1 h half life at 68°C. The activity of BGL was significantly enhanced by Fe^2+^ and Mn^2+^. The *V*_*max*_ of 64 U/mg and 120 U/mg were found for p-nitrophenyl-β-D-glucopyranoside (*K*_*m*_ value of 0.62 mM) and cellobiose (*K*_*m*_ value of 7.9 mM), respectively. It displayed high tolerance to glucose and cellobiose. The *K*_*cat*_ for cellobiose was 67.7 s^-1^ at 60°C and pH 6.4, when the concentration of cellobiose was 290 mM. It was activated by glucose at concentrations lower that 200 mM. With glucose further increasing, the enzyme activity of BGL was gradually inhibited, but remained 50% of the original value in even as high as 600 mM glucose.

**Conclusions:**

The article provides a useful novel β-glucosidase which displayed favorable properties: high glucose and cellobiose tolerance, independence of metal ions, and high hydrolysis activity on cellobiose.

## Introduction

Cellulosic biomass is the most abundant renewable resource on earth, whose natural degradation represents an important part of the carbon cycle within the biosphere [1]. β-Glucosidase (EC 3.2.1.21) is a glucosidase enzyme that acts upon β 1–4 bonds linking two glucose or glucose-substituted molecules. It is an important component of the cellulase enzyme system. The limiting step in the enzymatic saccharification of cellulosic material is the conversion of short-chain oligosaccharides and cellobiose, which was resulted from the synergistic action of endogucanases (EC 3.2.1.4) and cellobiohydrolases (EC 3.2.1.91), to glucose, a reaction catalyzed by β-glucosidases [2]. It is well established that cellobiose inhibits the activities of most cellobiohydrolases and endoglucanses [3]. β-glucosidases reduce cellobiose inhibition by hydrolyzing this disaccharide to glucose, thus allowing the cellulolytic enzymes to function more efficiently [4,5]*.* Furthermore, β-glucosidase is used as a flavor enzyme to enhance the flavor of wine, tea and fruit juice [6,7]. In fruits and other plant tissues many secondary metabolites, including flavor compounds, are accumulated in their glucosylated form [8,9]. Because β-glucosides constitute the majority of the known glycoconjugated flavor compounds, β-glucosidases play an important role in flavor liberation from these precursors. Therefore, producing high-activity and glucose-tolerant β-glucosidase has become important.

Recently, the search for β-glucosidases insensitive to glucose has increased significantly, for these enzymes would improve the process of saccharification of lignocellulosic materials. A few microbial β-glucosidases have been reported to tolerate glucose [10-14]. For example, β-glucosidases from *Aspergillus tubingensis* CBS 643.92, *A. oryzaeA. niger* CCRC 31494, *A. foetidus*, and marine microbial metagenome displayed high inhibition constant by glucose (*K*_*i*_) of 600 mM, 1390 mM, 543 mM, 520 mM, and 1000 mM, respectively. But these β-glucosidases have considerably lower specific activity for cellobiose than for p-nitrophenyl-β-D-glucopyranoside. Therefore, over-expression of thermostable β-glucosidase with high glucose tolerance and specific activity for cellobiose abilities will help shed light on degradation of cellulosic biomass.

Thermostable enzymes have several generic advantages, allowing a decreased amount of enzyme needed because of higher specific activity and elongated hydrolysis time due to higher stability. In addition, thermostable enzymes are generally more tolerant and allow more flexibility in process configurations [15,16]. Although some glucose-tolerant β-glucosidases from fungi and bacteria have been reported [10-14], the glucose-tolerant β-glucosidases genes have not been expressed and characterized from thermophilic bacteria. Bacterium *Thermoanaerobacterium thermosaccharolyticum* is a strict anaerobe that grows on wide range of hexose and pentose at temperature from 37°C to 75°C, which have attracted considerable interests to hydrogen production and thermostable enzyme production [17]. *T. thermosaccharolyticum* DSM 571 could utilize cellobiose, but the gene for β-glucosidase, the key enzyme in degradation cellobiose, was not reported in the Genbank (NC_014410.1). Because the optimal growth temperature for *T. thermosaccharolyticum* DSM 571 was at 60°C, the thermostable β-glucosidase could have a considerable potential for industrial applications. Owing to the inherent difficulty of cultivation of *T. thermosaccharolyticum* DSM 571, it is difficult to obtain a sufficient amount of cells for large-scale enzyme production. For the production of the recombinant protein, genetic engineering is the first choice because it is easy, fast, and cheap.

In this paper, we report the phylogenesis analysis, cloning, over-expression, and detailed biochemical characterization of the β-glucosidase from *T. thermosaccharolyticum* DSM 571. The favorable properties make the β-glucosidase a good candidate for utilization in biotechnological applications.

## Results

### Cloning and sequence analysis of *bgl*

**Figure 1:**
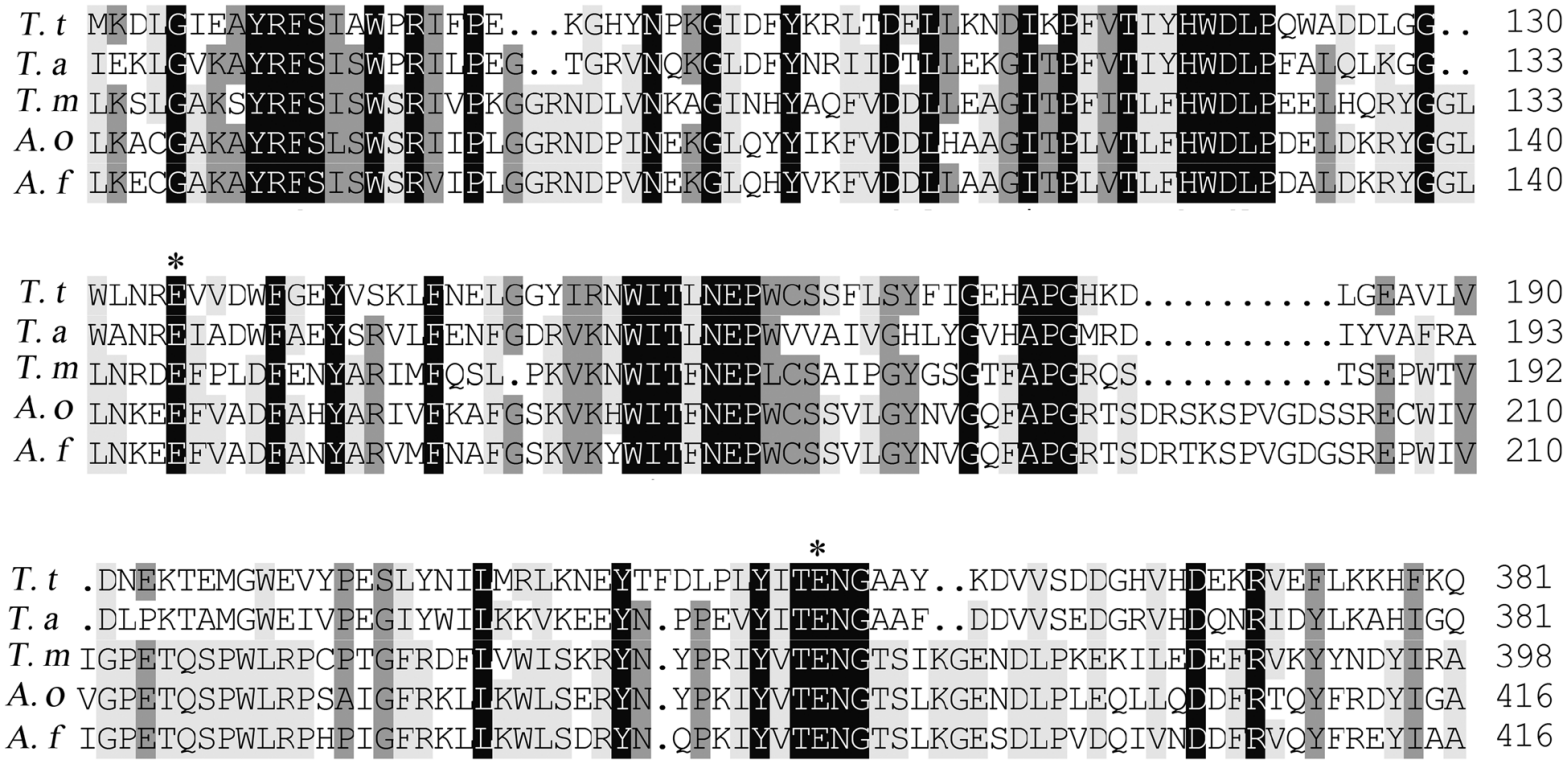
**Multiallignment of BGL with some GH1 family members.** Sequence alignment was performed by using Clustal X2.0. The active sites are indicated as* on the top of the alignment. *T. t*: *T. thermosaccharolyticum* DSM 571 (YP_003852393.1), *T. a*: *Trichoderma atroviride* (EHK41167.1), *T. m*: *Thermotoga maritima* (Q08638.1), *A. o*: *Aspergillus oryzae* (BAE57671.1), *A. f*: *Aspergillus fumigatus* (XP_752840.1).

By analysis of the genome sequence of *T. thermosaccharolyticum* DSM 571, a protein (Tthe_1813), defined as β-galactosidase in Genbank, consists of a 1,329-bp fragment encoding 443 amino acids, which belonged to family 1 of the glycoside hydrolases. It shares the highest sequence similarity of 66% with the β-glucosidses from *Thermoanaerobacter mathranii* (Genbank No. YP_003676178.1) and *Thermoanaerobacter pseudethanolicus* ATCC 33223 (Genbank No. YP_001665894.1), which were revealed by whole-genome sequencing but has not been biochemically characterized. Alignment of the BGL cluster with several representative members of GH1 indicated that they share similar blocks. The catalytic proton donor, Glu^135^ and Glu^351^ in BGL are well conserved among all GH1 proteins (Figure 1). The sequence around Glu^351^ in BGL is [LYT-NGAA], which is consistent with the consensus pattern of PS00572. The results indicated that the protein (Tthe_1813) could be a novel β-glucoside. Then the DNA fragment of a protein (Tthe_1813) gene was amplified from genomic DNA of *T. thermosaccharolyticum* DSM 571, and ligated to pET-20b at *Nde* I and *Xho* I sites to generate plasmid pET-20-BGL.

### Over-expression of BGL

**Figure 2:**
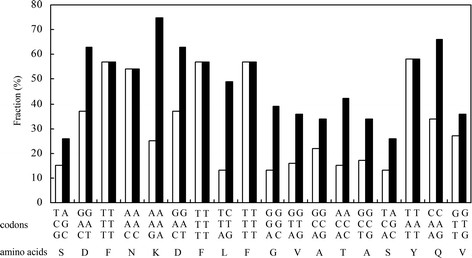
**The codons for the amino acid between the 1**^**st**^**and 19**^**th**^**which were subjected to site-directed mutagenesis.** Original sequence of the BGL (open square); optimal sequence of the BGL (filled square).

**Figure 3:**
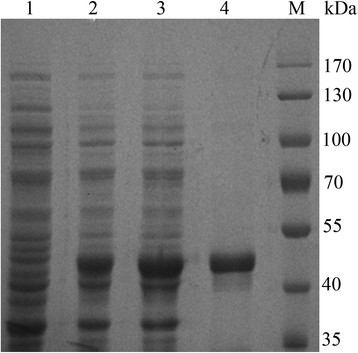
**SDS-PAGE analysis of recombinant BGL in*E. coli*JM109(DE3).** Lane M: protein marker, lane 1: cell-free extract of JM109(DE3) harboring pET-20b, lane 2: cell-free extract of JM109(DE3) harboring pET-20b-BGL, lane 3: cell-free extract of JM109(DE3) harboring pET-20b-BGLII, lane 4: purified BGL (4 μg).

In order to increase the expression level of BGL in *E. coli*, site-directed mutagenesis were designed and performed to optimize condons of BGL for *E. coli* expression system. pET-20-BGLII was obtained from pET-20-BGL in which the rare condons for the N-terminal amino acid residues were replaced by optimal codons in *E. coli* without and change of amino acid sequence (Figure 2), so pET-20-BGLII encodes the same β-glucosidase as that encoded by the wild-type gene. The β-glucosidase activity expression from pET-20-BGLII was 7.5 U/mL (11.2 U/mg total of cell protein) and was estimated to be about 30% of the total protein, which was about 1.7 times higher than the expressed from pET-20-BGL (Figure 3, lane 2 and 3).

### Purification and Characterization of recombinant BGL

The protein in the cell-free extract was purified to gel electro homogeneity after a heat treatment and a Ni-NTA affinity. The final preparation gave a single band on SDS-PAGE gel and the molecular mass of the enzyme was estimated to be 52 kDa (Figure 3, lane 4).

**Figure 4:**
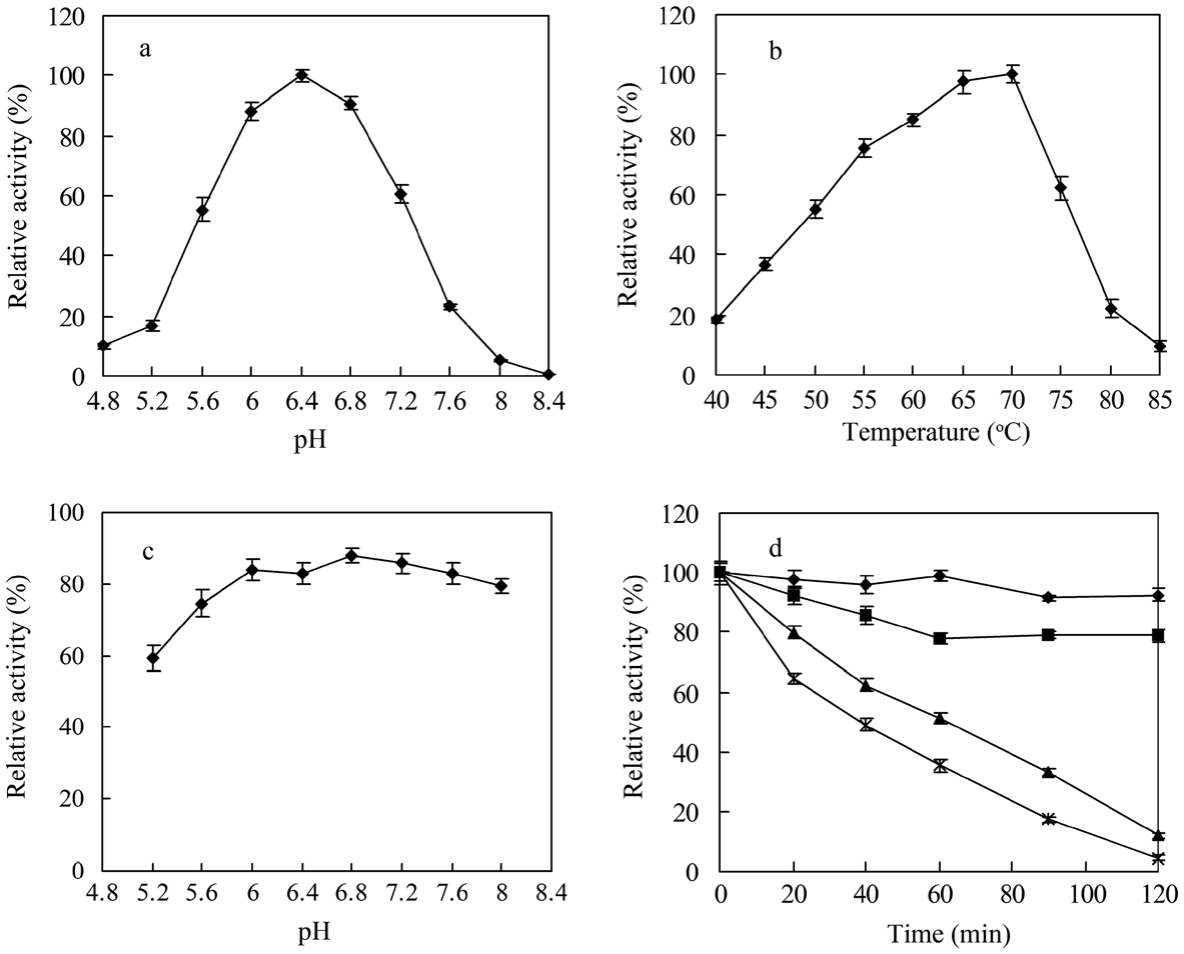
**The effects of pH and temperature on the activity and stability of the recombinant BGL.a** Effect of pH on BGL activity. **b** Effect of temperature on BGL activity. **c** The pH stability of the enzyme. **d** The thermostability of the BGL. The residual activity was monitored, while the enzyme was incubated at 50°C (*filled diamonds*), 65°C (*filled squares*), 68°C (*filled triangles*), and 70°C (*letter x*). The initial activity was defined as 100%.

The biochemical properties of BGL were investigated by using the purified recombinant BGL. The optimal pH of the BGL was determined to be 6.4 (Figure 4a), while the β-glucosidase activity was higher than 50% of the maximum activity at the pH range from 5.6 to 7.2. The enzyme was stable for about 1 h at pH 5.6 to 8.0 at 60°C in the absence of the substrate (Figure 4c). The optimal temperature of the BGL was 70°C, which the β-glucosidase activity was higher than 40% of the maximum activity at the temperature range from 45 to 75°C (Figure 4b). Thermostability assays indicated that its residual activity was more than 80% after being incubated at 60°C for 2 h (pH 6.4, Figure 4d).

The effects of metal ions and some chemicals on the enzyme activity were shown in (Table 1). In various assays, the enzyme activity was significantly enhanced by Fe^2+^, or Mn^2+^, and completely inactivated by Zn^2+^, Cu^2+^, Ag^2+^, or Hg^2+^. The effects of Mg^2+^, Ca^2+^, K^+^, Li^2^, or EDTA (10 mM) on the enzyme activity were not so significant.

**Table 1:** Effects of cations and reagents on purified BGL activity

Cation of reagent^a^	Residual activity (%)
Control	100
Fe^2+^	172
Mg^2+^	104
Zn^2+^	7
Mn^2+^	223
Ca^2+^	108
K^+^	101
Al^3+^	43
Li^+^	110
Cu^2+^	2
Hg^2+^	0
Co^2+^	37
Ag^2+^	19
EDTA (10 mM)	102

^a^ Final concentration, 1 mM or as indicated. Values shown are the mean of duplicate experiments, and the variation about the mean was below 5%.

### Effect of glucose on BGL activity and substrate specificity

**Figure 5:**
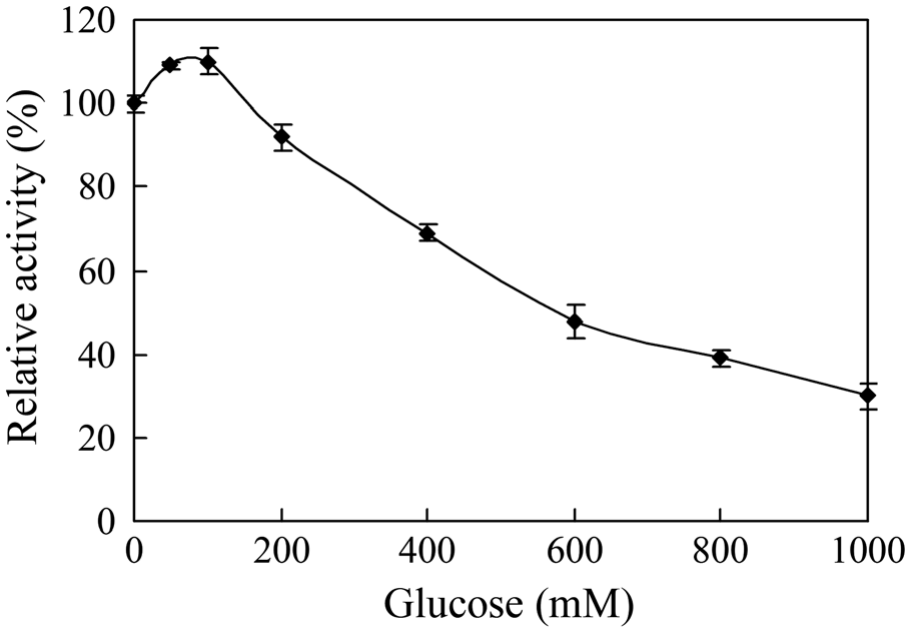
**The effects of glucose on BGL activity.** Influence of glucose on enzyme activity with p-nitrophenyl-β-D-glucopyranoside as the substrate.

The enzyme was able to hydrolyze p-nitrophenyl-β-D-glucopyranoside, cellobiose, and p-nitrophenyl-β-D-galactopyranoside, while no activity was detected upon p-nitrophenyl-α-L-arabinofuranoside, p-nitrophenyl-β-D-xylopyranoside, maltose, CMC, and sucrose. p-nitrophenyl-β-D-Galactopyranoside was hydrolyzed at 40% of that of p-nitrophenyl-β-D-glucopyranoside. The dependence of the rate of the enzymatic reaction on the substrates concentration followed Michaelis-Menten kinetics, with *K*_*m*_ and *V*_*max*_ values of 0.62 mM and 64 U/mg for p-nitrophenyl-β-D-glucopyranoside, and for cellobiose 7.9 mM and 120 U/mg under optimal conditions. The effects of the substrate, cellobiose (290 mM), on the enzyme activity were not significant. The *K*_*cat*_/*K*_*m*_ value for cellobiose 13.3 mM^-1^ s^-1^ was less than the β-glucosidase from *A. oryzae*, but the activity of β-glucosidase from *A. oryzae* was inhibited by cellobiose, and rapidly decreased above 50°C (Table 2). Furthermore, the enzyme activity was enhanced by the concentrations of glucose below 200 mM, and the enzyme activity was increased 110% when adding 100 mM glucose into reaction mixtures (Figure 5). When glucose was increased, the enzyme activity of BGL was gradually inhibited, with a *K*_*i*_ of 600 mM glucose (Figure 5). The properties of the glucose-tolerant β-glucosidase from other microorganisms are summarized in Table 2. As Table 2 shows, these enzymes have many distinct features, especially in their catalytic properties [12,13,18-21].

**Table 2:** **Characteristics of glucose-tolerant β-glucosidases from*T. thermosaccharolyticum*DSM 571 and other microorganisms**

Strain	*K*_*m*_ (mM)		*V*_*max*_ (U/mg)		*K*_*i*_ for glucose (mM)	Cellobiose inhibition (%)	*K*_*cat*_*/K*_*m*_ (mM^-1^ s^-1^) for cellobiose	Optimal Temp (°C)
	pNPG^a^	Cellobiose	pNPG	Cellobiose				
*T. thermosaccharolyticum*	0.63	7.9	64	120	600	No effect	13.3	70
Uncultured bacterium [13]	0.39	20.4	50.7	15.5	1000	ND^b^	0.65	40
*Debaryomyces vanrijiae*[18]	0.77	57.9	668	84.3	439	ND	2.43	40
*A. oryzae*[19]	0.55	7	1,066	353	1,390	50	36.1	50
*A. niger*[12]	21.7	ND	124.4	ND	543	ND	ND	55
*A. tubingensis*[10]	6.2	ND	28.4	0.32	600	ND	ND	60
*Candida peltata*[21]	2.3	66	108	8.5^c^	1400	No effect	0.1^c^	50
*Scytalidium thermophilum*[20]	0.29	1.61	13.27	4.12	>200	ND	1.7	60

^a^ pNPG: p-nitrophenyl-β-D-glucopyranoside.

^b^ ND: not determined.

^c^ It was calculated by the data based on the reference.

### Analysis of cellobiose degradation

**Figure 6:**
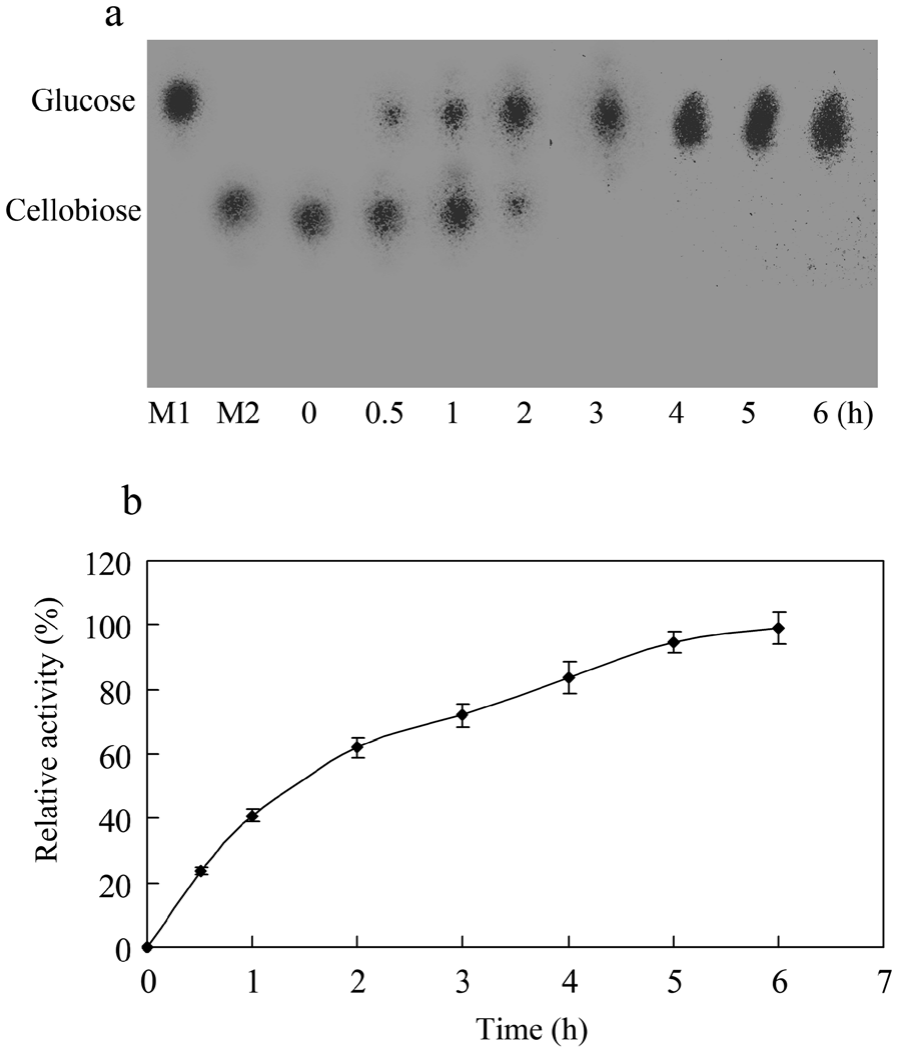
**Analysis of cellobiose hydrolysed by BGL.a** Thin-layer chromatography of the products from the reaction. M1: cellobiose, M2: glucose, lane 0.5, 1, 2, 3, 4, 5, 6: cellobiose (290 mM) incubated with BGL (1 μg) for different times. **b** the concentration of glucose analysis by HPLC.

Production of glucose from 290 mM cellobiose (10%) by the purified BGL was examined. Even if the final concentration of glucose in reaction reached about 580 mM, cellobiose (290 mM) was found to be degraded completely (Figure 6a, b). At the beginning of the reaction, the *K*_*cat*_ was 67.7 s^-1^ within one hour at 60°C which was identical to the theoretical value. During the whole degradation process, the *K*_*cat*_ was 28.2 s^-1^.

### Phylogenies analysis of BGL

**Figure 7:**
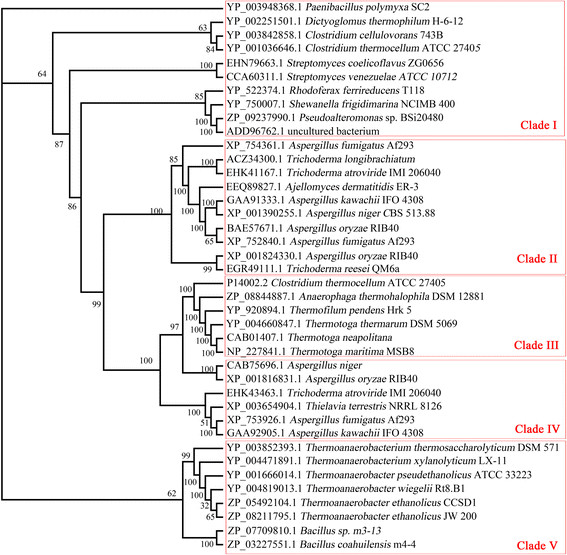
**The Neighbor-Joining (NJ) and Maximum-Parsimony (MP) trees results from analysis of β-glucosidases of 40 amino acid sequences.** Numbers on nodes correspond to percentage bootstrap values for 1000 replicates.

To gain insights into the evolutionary relationship among β-glucosidases, we constructed the phylogenetic trees of 40 candidate sequences using he NJ method and the MP method respectively, both supporting almost the same topology. The results revealed the presences of five well-supported clades: Clade II was GH1 β-glucosidases from fungi, and Clade III was the GH3 β-glucosidases from bacteria, and Clade IV was the GH3 β-glucosidases from fungi. The GH1 β-glucosidases from bacteria was divided into two clades: Clade I mainly contained mesophilic bacteria; Clade V mainly contained thermophile, which is formed by further divided into two subclades, of which one contains all thermophile, and the other *Bacillus* GH1 β-glucosidases. Clade II and clade III had a relatively close relationship, and the GH1 β-glucosidases from thermophile were distant from the other clades (Figure 7).

## Discussions

A classification of glycoside hydrolases based on amino acid sequence similarities was proposed a few years ago, wherein β-glucosidases were mainly grouped into two superfamilies of glycoside hydrolases I (GH1), and GH3 [22]. Although, the amino acid sequence analysis indicated that BGL belongs to GH1, it shared the highest sequence similarity of 66% with the β-glucosidses from *Thermoanaerobacter mathranii* (YP_003676178.1). Moreover, it shared only the 63% with the putative β-glucosidase (YP_004471891.1) the *Thermoanaerobacterium xylanolyticum* LX-11, both belonging to the genus *Thermoanaerobacterium*. The Phylogenies analysis showed that the BGL was distant with the glucose-tolerant β-Glucosidases from fungi and ADD96762.1 (Figure 7). The results indicated that the BGL could be a novel β-glucoside with some different properties. On the other hand, β-Glucosidases may be divided into three groups on the basis of their substrate specificity. The first group is known as aryl-β-glucosidases owing to strong affinity to aryl-β-glucose. The second group consists of cellobiases that hydrolyze oligosaccharides only. The third group is broad specific β-glucosidases that exhibit activity on a wide range of substrates, and are the most commonly observed form of β-glucosidases [23]. The BGL, which was high affinity to p-nitrophenyl-β-D-glucopyranoside, hydrolyzed cellobiose, p-nitrophenyl-β-D-glucopyranoside, and p-nitrophenyl-β-D-galactopyranoside, but not p-nitrophenyl-α-L-arabinofuranoside, p-nitrophenyl-β-D-xylopyranoside, maltose, sucrose, and CMC. These results indicated that BGL belonged to the first group.

Enzymatic hydrolysis of cellulose is a complex process, the last step being a homogenous catalysis reaction involving the action of β-glucosidase on cellobiose. Cellobiose is a strong inhibitor of both cellobiohydrolases and endocellulases. Therefore, β-glucosidase with high tolerance for glucose has become heated in these fields. Fungi, especially *Aspergillus* species, are generally considered to be a good producer with high yield of β-glucosidases [24]. But the major β-glucosidases belonging to family 3 of the glycoside hydrolases (GH3) from *Aspergillus* species were subject to competitive inhibition of glucose to produce glucose, the *K*_i_ is generally 1–20 mM [10,14]. The minor β-glucosidases, which molecular weights are 40–50 kDa, exhibited a tolerance to glucose (Table 2). The effect of glucose on the BGL activity revealed that the enzyme is not only resistant to end-product inhibition, but is activated by glucose at concentrations from 0 to 0.2 M. Only two β-glucosidases, activated by glucose, have been reported from *Scytalidium thermophilum* and marine microbial (Table 2) [13,20].

Moreover, high specific activity for cellobiose and tolerance to substrate inhibition are other advantages for β-glucosidase in enzymatic hydrolysis of cellulose. Although, several β-glucosidases from a few fungi and bacteria show high glucose tolerant with *K*_*i*_ values of more than 200 mM, the *V*_*max*_ values of these enzymes for cellobiose were much lower than for p-nitrophenyl-β-D-glucopyranoside. The *V*_*max*_ value of BGL for cellobiose was 120 U/mg, which was about 2 times higher than the *V*_*max*_ value of BGL for p-nitrophenyl-β-D-glucopyranoside. To our knowledge, in only one other study have workers described the purification and characterization (from *A. oryzae*) of a β-glucosidase having such a high tolerance to glucose and high specific activity for cellobiose [19]. But the specific activity of β-glucosidase from *A. oryzae* for cellobiose was much lower than for p-nitrophenyl-β-D-glucopyranoside (Table 2). The BGL was only the β-glucosidase been reported that it is not only resistant to glucose, but had higher specific activity for cellobiose than for p-nitrophenyl-β-D-glucopyranoside. In addition, the BGL had high tolerance to substrate inhibition, cellobiose. The *K*_*cat*_ of BGL was 67.7 s^-1^ at 60°C and pH 6.4, when the concentration of cellobiose was 10% (Table 2).

The chemical agents had various effects on the activity of BGL. The chelating agent EDTA displayed no influence on the β-glucosidase activity, indicating that the β-glucosidase is not a metalloprotein. However, the β-glucosidase activity was greatly stimulated by Fe^2+^ or Mn^2+^, which implied that Fe^2+^ or Mn^2+^ is required for the maximal activity of BGL. These results distinguish BGL from the other bacteria β-glucosidases, on which Ca^2+^ show positive effects [13]. In practical applications, the high thermostability of the enzyme is desired because the longer active life means the less consumption of the enzyme. The BGL residual activity was more than 80% after being incubated at 60°C for 2 h, and it in enzymatic hydrolysis of cellulose exhibited high activity in broad temperature, which could keep at high levels at temperatures from 45 to 70°C.

The properties of the BGL demonstrated a great potential of the gene in the genetic modification of strains for biomass degradation. Differences in codon usage preference among organisms lead to a variety of problems concerning heterologous gene expression, which can be overcome by rational gene design and gene synthesis. Protein with multiple repetitive rare codons especially within the first 20 amino acids of the amino terminus of the protein may significantly reduce the protein expression. Sometimes, it shuts down the expression completely. Since the rare codons of *bgl* from 1–20 amino acids were all changed into optimized codons, the activity of BGL was increased by about 70% (Figure 3). More optimization of codons for the other amino acid residues in the ORF of *bgl* may give further improvement in the gene expression levels.

## Conclusion

With this study, we successfully over-expressed the novel β-glucosidase (BGL) gene *bgl* from *T. thermosaccharolyticum* DSM 571 by replacing the rare codons with the optimal codons in *E. coli*. The Phylogenies analysis showed that the BGL had close relationship with the β-Glucosidases from thermophile, and was distant from the other glucose-tolerant β-Glucosidases. As compared on the enzyme properties, the BGL was higher tolerant to glucose and cellobiose, more efficient in hydrolysis of cellibiose, more thermal stability than β-glucosidases from other microorganisms. Thus, this study provides a useful novel β-glucosidase, which may be used to improve the enzymatic conversion of cellulosic to glucose through synergetic action.

## Materials and Methods

### Bacterial Strains, Plasmids, Growth Media

*Thermoanaerobacterium thermosaccharolyticum* DSM 571 was purchased from DSMZ (http://www.dsmz.de). It was grown anaerobically at 60°C as described previously [17]. *Escherichia coli* JM109 and JM109(DE3) was grown at 37°C in Luria-Bertani medium (LB) and supplemented with ampicillin when required. The expression vectors pET-20b (Novagen) were employed as cloning vector and expression vector.

### DNA manipulation

DNA was manipulated by standard procedures [25]. QIAGEN Plasmid Kit and QIAGEN MinElute Gel Extraction Kit (Qiagen, USA) were employed for the purification of plasmids and PCR products. DNA restriction and modification enzymes were purchased form TaKaRa (Dalian, China). DNA transformation was performed by electroporation using GenePulser (Bio-Rad, USA). Site-directed mutagenesis of genes and the modification of the plasmids were performed by inverse-PCR followed by phosporylation and self-ligation using T4 polynucleotide kinase and T4 DNA ligase.

### Plamid constructions

The β-glucosidase gene *bgl* was amplified from *T. thermosaccharolyticum* DSM 571 genomic DNA by PCR using primers bgl-1 and bgl-2 (Table 3), the PCR products were digested with *Nde* I and *Xho* I and inserted into pET-20b at *Nde* I and *Xho* I sites, yielding the plasmid pET-20-BGL.

**Table 3:** Nucleotide sequences of used primers

Primer	Nucleotide sequence
bgl-1	CCCCATATGTCGGACTTTAACAAGGAC
bgl-2	CCCCTCGAGAATGGTCCTAGTGGAAATAAG
bgl-3	TTTGG***C***GT***G***GC***G***AC***C***GC***GAGC***TATCA***G***GT***G***GAAGG TGCTTACAATGAGGA
bgl-4	***C***A***G***AAA***A***TC***T***TTGTTAAA***A***TC***GCT***CATATGTATATCT CCTTCTTAAAG

The boldface italic nucleotides represented mutations for optimizing codons.

In order to improve the expression level of recombinant BGL, the internal region from 1^st^ to 19^th^ amino acids in open reading frame of *bgl* was mutated in situ by inverse-PCR to replace the rare codons with the optimal codons of *E. coli*; the primers for the inverse-PCR were designated as bgl-3 and bgl-4 (Table 3). Inverse-PCR with primers was carried out using Pyrobest with pET-20-BGL as template, generating the plasmid pET-20-BGLII.

### Expression and purification of BGL

Plasmids pET-20-BG and pET-20-BGLII were transformed into *E. coli* JM109(DE3), and induced to expressed recombinant BGL by adding isopropyl-β-D-thiogalactopyranoside (IPTG) to final concentration of 0.8 mM at OD_600_ about 0.7, and incubated further at 30°C for about 6 h.

One liters of the recombinant cells carrying pET-20-BGLII were harvested by centrifugation at 5,000 g for 10 min at 4°C, and washed twice with distilled water, resuspended in 50 mL of 5 mM imidazole, 0.5 mM NaCl, and 20 mM Tris–HCl buffer (pH 7.9), and French-pressured for three times. The cell extracts were heat treated (60°C, 30 min), and then cooled in an ice bath, and centrifuged (20,000 g, 4°C, 30 min). The resulting supernatants were loaded on to an immobilized metal affinity column (Novagen, USA), and eluded with 1 M imidazole, 0.5 M NaCl, and 20 mM Tris–HCl buffer (pH 7.9). Protein was examined by SDS-PAGE [26], and the protein bands were analyzed by density scanning with an image analysis system (Bio-Rad, USA). Protein concentration was determined by the Bradford method using BSA as a standard.

### Determination of enzyme activities and properties

The reaction mixture, containing 50 mM imidole-potassium buffer (pH 6.4), 1 mM p-nitrophenyl-β-D-glucopyranoside, and certain amount of β-glucosidase in 0.2 mL, was incubated for 5 min at 70°C. The reaction was stopped by adding 1 mL of 1 M Na_2_CO_3_. The absorbance of the mixture was measured at 405 nm. One unit of enzyme activity was defined as the amount of enzyme necessary to liberate 1 μmol of *p*NP per min under the assay conditions.

The optimum pH for activity β-glucosidase was determined by incubation at 70°C for 5 min in the 50 mM imidole-potassium buffer from pH 4.8 to 8.4. The optimum temperature for the enzyme activity was determined by standard assay ranging from 45 to 85°C in the 50 mM imidole-potassium buffer, pH 6.0. The results were expressed as percentages of the activity obtained at either the optimum pH or the optimum temperature.

The pH stability of the enzyme was determined by measuring the remaining activity after incubating the enzyme (0.1 μg) at 50°C for 1 h in the 50 mM imidole-potassium buffer from pH 5.2 to 8.0. To determine the effect of temperature on the stability of BGL, the enzyme (0.1 μg) in the 50 mM imidole-potassium buffer (pH 6.4) was pre-incubated for various times at 50°C, 65°C, 68°C and 70°C in the absence of the substrate. The activity of the enzyme without pre-incubation was defined as 100%.

The effects of metals and chemical agents on β-glucosidase activity of purified enzyme (0.1 μg) were determined. Fe^2+^, Mg^2+^, Zn^2+^, Mn^2+^, Ca^2+^, K^+^, Al^3+^, Li^2+^, Cu^2+^, Co^2+^, and Hg^2+^ were assayed at concentrations of 1 mM in the reaction mixture. The chemical agents EDTA (10 mM) were assayed. The enzyme was incubated with each reagent for 10 min at 50°C before addition of p-nitrophenyl-β-D-glucopyranoside to initiate the enzyme reaction. Activity was determined as described above and was expressed as a percentage of the activity obtained in the absence of the chemical agents and metal cations.

The substrate specificity of the enzyme (0.1 μg) was tested by using following p-nitrophenyl-β-D-glucopyranoside, p-nitrophenyl-β-D-xylopyranoside, p-nitrophenyl-α-L-arabinofuranoside, maltose, sucrose, and cellobiose. Kinetic constant of BGL was determined by measuring the initial rates at various p-nitrophenyl-β-D-glucopyranoside concentrations (0.2, 0.4, 0.6, 0.8, 1, 2, and 4.0 mM) or various cellobiose concentration (2, 4, 6, 8, 10, 12, 14, and 16 mM) under standard reaction conditions. The *K*_*i*_ value of glucose was defined as amount of glucose required for inhibiting 50% of the β-glucosidase activity and was given as the averages of three separate experiments performed in duplicate.

### Phylogenies analysis of BGL

The condon usage preference of *E. coli* in translation initiation region of pET-20-BGL was analyzed by using codon usage tool (http://gcua.schoedl.de/). The potential ORF of *bgl* was searched using the ORF search tool provided by the National Center for Biotechnology Information (http://www.ncbi.nlm.nih.gov). Database searching was performed with Blast at NCBI and against CAZy (http://www.cazy.org). The active site of the enzyme was analyzed with the prosite tool (http://prosite.expasy.org/scanprosite). The multiple sequence alignment tool Clustal X2.0 was used for multiple protein sequence alignment [27]. Sequences were further edited and aligned manually, when necessary, using the Mega 5 for editing. For phylogenetic analyses of conserved domains, sequences were trimmed so that only the relevant protein domains remained in the alignment [28]. Phylogenetic relationships were inferred using the Neighbor-Joining (NJ) and Maximum-Parsimony (MP) method as implemented in Paup 4.0 for the NJ and MP trees, the results were evaluated with 1000 bootstrap replicates [29]. The generated trees were displayed using TREEVIEW 1.6.6 (http://taxonomy.zoology.gla.ac.uk/rod/treeview.html).

### Analysis of cellobiose degradation

The cellobiose was treated with purified BGL, and the degradation was subjected to analysis of thin-layer chromatography (TLC) and HPLC. The reaction mixture (20 μL) contained 290 mM cellobiose, and 1 μg of BGL in 50 mM imidole-potassium buffer (pH 6.4). The reaction was performed for various times at 60°C, and stopped by heating for 5 min in a boiling water bath. After centrifuged for 10 min at 10,000 g, supernatants of the reaction mixtures were applied on silica gel TLC plates (60F254, Merck Co.). Sugars on the plates were partitioned with a solvent system consisting of *n*-butanol, acetic acid, and water (2:1:1, by vol/vol), and detected using the orcinol reagent [30]. The concentration of glucose was examined by HPLC on a carbohydrate analysis column (Waters Sugarpak1, USA) with water as a mobile phase.

## Misc

Jianjun Pei and Qian Pang contributed equally to this work

## Competing interests

The authors declare that they have no competing interests.

## Authors’ contributions

JP carried out the cloning and over-expression and drafted the manuscript. QP and SF helped to purify and characterize the BGL. LZ directed the over-all study and drafted the manuscript. HS helped to perform phylogenies analysis of β-glucosidases. All authors read and approved the final manuscript.

## References

[B1] Bhat MK Cellulases and related enzymes in biotechnology Biotechnol Adv 2000 18 355 383 10.1016/S0734-9750(00)00041-0 14538100

[B2] Brethauer S Wyman CE Review: continuous hydrolysis and fermentation for cellulosic ethanol production Bioresour Technol 2010 101 4862 4874 10.1016/j.biortech.2009.11.009 20006926

[B3] George SP Ahmad A Rao MB Studies on carboxymethyl cellulose produced by an alkalothermophilic actinomycete Bioresour Technol 2001 77 171 175 10.1016/S0960-8524(00)00150-4 11272024

[B4] Shin HJ Yang JW Galactooligosaccharide synthesis from lactose byPenicillium Funiculosumcellulase Biotechnol Lett 1996 18 143 144 10.1007/BF00128668

[B5] Chauve M Mathis H Huc D Casanave D Monot F Ferreira N Comparative kinetic analysis of two fungal β-glucosidases Biotechnol Biofuels 2010 3 3 10.1186/1754-6834-3-3 20181208 PMC2847552

[B6] Delcroix A Günata Z Sapis JC Salmon JM Bayonove C Glycosidase activities of three enological yeast strains during wine making: Effect on the terpenol content of Muscat wine Am J Enol Vitic 1994 45 291 296

[B7] Engel KH Tressl R Formation of aroma components form on volatile precursors in passion fruit J Agric Food Chem 1983 31 998 1002 10.1021/jf00119a019

[B8] Song X Xue Y Wang Q Wu X Comparison of three thermostable β-glucosidases for application in the hydrolysis of soybean isoflavone glycosides J Agric Food Chem 2011 59 1954 1961 10.1021/jf1046915 21294581

[B9] Williams PJ Christopher RS Bevan W Massy-Westropp RA Studies on the hydrolysis of vitis vinifera monoterpenne precursor compounds and model monoterpene β-D-glucosides rationalizing the monoterpene composition of grapes J Agric Food Chem 1982 30 1219 1223 10.1021/jf00114a054

[B10] Decker CH Visser J Schreier P β-glucosidase multiplicity fromAspergillus tubingensisCBS 643.92: purification and characterization of four β-glucosidases and their differentiation with respect to substrate specificity, glucose inhibition and acid tolerance Appl Microbiol Biotechnol 2001 55 157 163 10.1007/s002530000462 11330708

[B11] Saha BC Bothast RJ Production, purification, and characterization of a highly glucose-tolerant novel β-Glucosidase fromCandida peltata Appl Environ Microb 1996 62 3165 3170 10.1128/aem.62.9.3165-3170.1996 PMC168111 8795205

[B12] Yan TR Liau JC Purification and characterization of a glucose-tolerant β-glucosidase fromAspergillus nigerCCRC 31494 Biosci Biotech Biochem 1997 61 965 970 10.1271/bbb.61.965 9214755

[B13] Fang Z Fang W Liu J Hong Y Peng H Zhang X Sun B Xiao Y Cloning and characterization of a β-glucosidase from marine microbial metagenome with excellent glucose tolerance J Microbiol Biotechnol 2010 20 9 1351 1358 10.4014/jmb.1003.03011 20890102

[B14] Decker CH Visser J Schreier P β-glucosidases from five blackAspergillus species: study of their physico-chemical and biocatalytic properties J Agric Food Chem 2000 48 4929 4936 10.1021/jf000434d 11052758

[B15] Viikari L Alapuranen M Puranen T Vehmaanperä J Siika-aho M Thermostable enzymes in lignocellulose hydrolysis Adv Biochem Eng Biotechnol 2007 108 121 145 17589813 10.1007/10_2007_065

[B16] Zhang J Siika-aho M Puranen T Tang M Tenkanen M Viikari L Thermostable recombinant xylanases fromNonomuraea flexuosaandThermoascus aurantiacusshow distinct properties in the hydrolysis of xylans and pretreated wheat straw Biotechnol Biofuels 2011 4 12 10.1186/1754-6834-4-12 21592333 PMC3114720

[B17] Suphavadee C Tachaapaikoon C Pason P Kyu KL Kosugi A Mori Y Ratanakhanokchai K Isolation and characterization of endocellulase-free multienzyme complex from newly isolatedThermoanaerobacterium thermosaccharolytiumstrain NOI-1 J Microbiol Biotechnol 2011 21 3 284 292 21464600

[B18] Belancic A Gunata Z Vallier MJ Agosin E β-Glucosidase from the grape native yeastDebaryomyces Vanrijiae: Purification, characterization, and its effect on monoterpene content of a muscat grape juice J Agric Food Chem 2003 51 1453 1459 10.1021/jf025777l 12590497

[B19] Riou C Salmon JM Vallier MJ Günata Z Barre P Purification, characterization, and substrate specificity of a novel highly glucose-tolerant β-glucosidase fromAspergillus oryzae Appl Environ Microbiol 1998 64 3607 3614 9758774 10.1128/aem.64.10.3607-3614.1998 PMC106471

[B20] Zanoelo FF Polizeli ML Terenzi HF Jorge JA β-Glucosidase activity from the thermophilic fungusScytalidium thermophilumis stimulated by glucose and xylose FEMS Microbiol Lett 2004 240 137 143 10.1016/j.femsle.2004.09.021 15522500

[B21] Saha BC Bothast RJ Production, purification, and characterization of a highly glucose-tolerant novel β-glucosidase fromCandida petltata Appl Environ Microbiol 1996 62 3165 3170 8795205 10.1128/aem.62.9.3165-3170.1996 PMC168111

[B22] Henrissat B Bairoch A Updating the sequencebased classification of glycosyl hydrolases Biochem J 1996 316 695 696 8687420 10.1042/bj3160695 PMC1217404

[B23] Rojas A Arola L Romeu A β-glucosidase families revealed by computer analysis of protein sequences Biochem Mol Biol Int 1995 35 1223 1231 7492960

[B24] Wen Z Liao W Chen S Production of cellulase/β-glucosidase by the mixed fungi culture and on dairy manure Process Biochem 2005 40 3087 3094 10.1016/j.procbio.2005.03.044 15917591

[B25] Sambrook J, Fritsch EF, Maniatis T Molecular cloning: a laboratory manual 1989 Cold Spring Harbor Laboratory Press, Cold Spring Harbor, NY

[B26] Laemmli UK Cleavage of structural proteins during the assembly of the head of bacteriophage T4 Nature 1970 227 680 685 10.1038/227680a0 5432063

[B27] Larkin MA Blackshields G Brown NP Clustal W and clustal X version 2.0 Bioinformatics 2007 23 2947 2948 10.1093/bioinformatics/btm404 17846036

[B28] Tamura K Peterson D Peterson N Stecher G Nei M Kumar S MEGA5: molecular evolutionary genetics analysis using maximum likelihood, evolutionary distance, and maximum parsimony methods Mol Biol Evol 2011 28 2731 2739 10.1093/molbev/msr121 21546353 PMC3203626

[B29] Wilgenbusch JC Swofford D Inferring evolutionary trees with PAUP Curr Protoc Bioinformatics 2003 http://www.currentprotocols.com/protocol/bi0604 Chaper 6, unit 6.4. 10.1002/0471250953.bi0604s00 18428704

[B30] Okuyama M Okuno A Shimizu N Mori H Kimura A Chiba S Carboxyl group of residue Asp647 as possible proton donor in catalytic reaction of α-glucosidase fromSchizosaccaromyces pombe Eur J Biochem 2001 268 2270 2280 10.1046/j.1432-1327.2001.02104.x 11298744

